# Measuring the effects of misinformation exposure and beliefs on behavioural intentions: a COVID-19 vaccination study

**DOI:** 10.1186/s41235-022-00437-y

**Published:** 2022-10-01

**Authors:** Constance de Saint Laurent, Gillian Murphy, Karen Hegarty, Ciara M. Greene

**Affiliations:** 1 School of Psychology, University College Dublin, Dublin, Ireland; 2 School of Applied Psychology, University College Cork, Cork, Ireland

**Keywords:** Misinformation, Fake news, Vaccine, COVID-19

## Abstract

Misinformation has been a pressing issue since the beginning of the COVID-19 pandemic, threatening our ability to effectively act on the crisis. Nevertheless, little is known about the actual effects of fake news on behavioural intentions. Does exposure to or belief in misinformation about COVID-19 vaccines affect people’s intentions to receive such a vaccine? This paper attempts to address this question via three preregistered experiments (N = 3463). In Study 1, participants (*n* = 1269) were exposed to fabricated pro- or anti-vaccine information or to neutral true information, and then asked about their intentions to get vaccinated. In Study 2, participants (*n* = 646) were exposed to true pro- and anti-vaccine information, while Study 3 (*n* = 1548) experimentally manipulated beliefs in novel misinformation about COVID-19 vaccines by increasing exposure to the information. The results of these three studies showed that exposure to false information about the vaccines had little effect on participants’ intentions to get vaccinated, even when multiple exposures led them to believe the headlines to be more accurate. An exploratory meta-analysis of studies 1 and 3, with a combined sample size of 2683, showed that exposure to false information both supporting and opposing COVID-19 vaccines actually *increased* vaccination intentions, though the effect size was very small. We conclude by cautioning researchers against equating exposure to misinformation or perceived accuracy of false news with actual behaviours.

**Supplementary Information:**

The online version contains supplementary material available at 10.1186/s41235-022-00437-y.

## Significance statement

The advent of the COVID-19 pandemic has drawn additional attention to the problem of online misinformation. This is particularly evident when we consider the potential consequences of misinformation for important health behaviours such as vaccination. It may therefore be surprising to learn that there is little evidence available about the direct effects of misinformation exposure on behaviour, as most research has focussed on belief in or willingness to share “fake news”. In this paper, we describe three experiments evaluating the effects of exposure to pro- and anti-vaccine information on participants’ intention to get a COVID vaccine. We report that a single exposure to a piece of true or false information about vaccination did not significantly affect participants’ willingness to get vaccinated. In Experiment 3, we report that showing participants the same piece of misinformation on multiple occasions increased their belief in the information, but still did not affect their behavioural intentions. Our results suggest that the relationship between exposure to, belief in and behavioural response to fake news is not straightforward. It is critical to understand when and how misinformation might affect individual or public behaviour, so that efforts to counteract it can be targeted where they are needed.

## Introduction

While the COVID-19 pandemic has undeniably been accompanied by an impressive amount of misinformation—spreading faster and further than the disease itself (Depoux et al., 2020)—it is difficult to estimate the scale of the issue. There are, however, indirect signs: Between January and March 2020, for instance, the number of COVID-19 fact-checks available in English increased by 900% (Brennen et al., 2020). On Twitter, analyses of the URLs in COVID-related tweets have also shown that unreliable websites receive more attention than high-quality health websites, although mainstream media retains the lion’s share (Singh et al., 2020). More worryingly, studies have shown that endorsement of misinformation is correlated with lower adherence to safety guidelines and reduced vaccination intentions (e.g. Earnshaw et al., 2020; ), as well as increased intentions to use unproven treatments (e.g. Bertin et al., 2020; Teovanović et al., 2021).

In this context, it is often assumed that the relationships between exposure to misinformation, inaccurate beliefs and behaviour are causal. It makes intuitive sense that being exposed to fake news would make it more likely that an individual will believe the misinformation contained in it and act accordingly, but is it really that easy to convince people to refuse a vaccine or to take an unproven treatment like Ivermectin? Decades of research on persuasion, attitudes, and behaviour have demonstrated that persuasion is difficult, and the relationship between attitudes and behaviour is complicated at best (Crano & Prislin, 2006; Wood, 2000). Surprisingly, very few studies have looked at the effects of misinformation exposure on behaviours, beyond intentions to share information on social media. While sharing behaviours certainly contribute to the spread of fake news, it does not follow that such news is always taken literally or acted upon. People share misinformation for all kinds of reasons—from a desire to warn or educate others to signalling political ideology—and comments on false news shared on social media show that it is most often disbelieved (Metzger et al., 2021).

To the best of our knowledge, only two studies have explored the consequences of fake news on real-world behaviours, both by looking at geographical patterns. Cantarella et al. (2019) used linguistic differences in South Tyrol in Italy to estimate how much misinformation the inhabitants of specific geographical areas were exposed to and whether it led to populist voting. They concluded that fake news did not significantly affect group-level voting behaviours. Forati and Ghose (2021) explored the relation between geo-localised Twitter data and county-level COVID-19 incidence rates in the USA. They found that most epidemic peaks were accompanied by peaks in coronavirus-related online activity, and that counties that saw more fake news being shared struggled the most to implement necessary restrictions. This led them to conclude that misinformation did affect health-related behaviours. However, in both cases it is difficult to assess the extent to which individuals were actually exposed to fake news, and whether there were any confounding factors. For instance, it is possible that areas where more fake news about COVID-19 were shared simply had more inhabitants inclined to believe that the virus was not particularly dangerous. This could have made them more likely to both share fake news about the pandemic—often presenting the situation as blown out of proportion—and to later refuse public health measures, without one necessarily causing the other.

Other studies have turned to experiments, looking at the effects of misinformation exposure on behavioural intentions. The theory of planned behaviour (Ajzen, 1991) holds that behavioural intention is an essential precursor to action, along with perceived behavioural control. In practice, reported intentions to engage in a behaviour are strong (but not perfect) predictors of actual behaviour. This has been observed across a range of domains, including alcohol consumption (Cooke et al., 2016), attendance at health screening programmes (Cooke & French, 2008) and engagement in pro-environmental behaviour (Bamberg & Moser, 2007). Thus, misinformation that has a clear influence on behavioural intentions may be reasonably expected to also affect real-world behaviour. Jolley and Douglas (2014) proposed one of the first studies of this kind, showing that participants presented with conspiracy theories on vaccines reported reduced intentions to vaccinate a fictitious child than those presented with factual information. More recently, three studies have looked at exposure to COVID-19 and behavioural intentions. First, Loomba et al. (2021), in the largest study of its kind (*N* = 8001), found that participants exposed to fake news about COVID-19 vaccines were less likely to report an intention to get vaccinated. Importantly, data were collected for this study in September 2020, while the COVID-19 vaccines were still in development and several months before they were approved and made available to the public. Participants’ decisions about whether or not to get a COVID vaccine were therefore hypothetical at this stage, and they may have responded differently once accurate information about specific vaccines was available. Moreover, it is difficult to rule out the possibility that the design of the study affected the responses. Participants were shown five fake news items, and after each one were asked, (1) whether it made them less inclined to be receive a COVID-19 vaccine, (2) if they agreed with the item, (3) if they found it trustworthy, (4) if they were likely to check its accuracy, and (5) if they were likely to share it. Research has shown that asking about accuracy changes how headlines are considered (Pennycook et al., 2020). It is therefore possible that repeatedly asking participants how information (which they did not know was false) would affect their intentions to get vaccinated might have contributed to the effects observed by the researchers.

Second, MacFarlane et al. (2021) studied the effects of exposure to misinformation about a fake COVID-19 treatment, vitamin E, on the participants’ willingness to pay for that treatment (*N* = 678). They found that such exposure did not affect how much the participants were ready to pay for vitamin E compared to a control group who were shown non-coronavirus-related messages supporting vitamin E. This study was designed to evaluate the effects of two debunking interventions, not to measure the effects of fake news, and indeed, the authors found that participants in the debunking conditions were willing to pay less for vitamin E than those in the misinformation condition. Unfortunately, it is difficult to conclude whether misinformation affected the participants’ choices in this study, as the control condition included exposure to accurate information supporting the use of the false treatment in conditions unrelated to COVID-19.

Finally, Greene and Murphy (2021) measured the effects of exposure to novel fake news stories on the participants’ intentions to engage in related behaviours (*N* = 3746); for instance, showing participants a headline about how caffeine could help reduce severe coronavirus symptoms and comparing their intentions to drink more coffee in the future with a control group. Some headlines seemed to affect intentions, albeit with a small effect; for example, exposure to a false headline about a contact tracing app being used to monitor people’s activities led to a 5% decrease in intentions to download the app. Others, such as a false headline describing a conspiracy relating to COVID-19 vaccines, did not result in a significant change in vaccination intentions. While these results may seem inconsistent, they are in line with the overall mixed results obtained by the few studies on the topic.

If research on misinformation and its effects on behavioural intentions is still in its infancy, investigations of the effects of information exposure on attitudes and behavioural change are well-established and are fundamental to the literature on persuasion and attitude change in social psychology. In a recent review, Albarracin and Shavitt (2018) reported that exposure to information across a range of topics, including interventions to reduce risky sexual behaviour and alcohol and drug use, typically displays small-medium effects on participant attitudes, with an average effect of approximately *d* = 0.20. Attitudinal change sometimes (but not always) leads to behaviour change (see Verplanken & Orbell, 2022 for a review).

The relationship between intention and behaviour is complex and requires further investigation (Dai & Albarracín, 2022). Nevertheless, extant evidence suggests that the effect of information exposure on attitude and behaviour change can be moderated by appeals to strong emotions such as fear and anger (Lambert et al., 2010; Tannenbaum et al., 2015), a prominent feature of much “fake news” (Ghanem et al., 2020; Vosoughi et al., 2018). In this context, it is not clear whether misinformation presented in a given format (e.g. a news headline) should be expected to have clear effects on subsequent behavioural intentions. While the persuasion literature has not typically focussed on information in the form of headlines, there are ample investigations of short-form interventions, such as public health posters, social media posts and media advertisements aimed at reducing unhealthy behaviours (e.g. smoking or excessive alcohol consumption; Etter & Laszlo, 2005; Loman et al., 2018) or increasing health-promoting behaviours (e.g. attendance at cancer screening appointments or sunscreen usage; Brouwers et al., 2011; Plackett et al., 2020; Smith et al., 2002). Some of these interventions have been more successful than others, but in principle there is nothing to suggest that brief manipulations cannot affect behaviour.

### The present studies

The aim of this paper is to add to the literature on the effects of fake news exposure by focusing on the pressing issue of COVID-19 vaccines. We report three preregistered experiments looking at the effects of exposure to false or accurate information about COVID-19 vaccines on intentions to get vaccinated. Exposure to misinformation is often more limited than public discourse would have us believe; Americans saw an average of just 1.14 fake news stories during the 2016 presidential campaign (Allcott & Gentzkow, 2017). Thus, studies 1 and 2 presented participants with a single exposure to a piece of pro- or anti-vaccination information and examined the effects of this information on behavioural intentions.

In Study 1, unvaccinated participants were presented with false information on the vaccines, either supporting or opposing their use, while the control group was presented with true, neutral information about the pandemic. They were then asked about their intentions to get vaccinated against COVID-19, alongside a few other behavioural intentions. The analyses show that the experimental conditions did not affect the participants’ intentions to receive a COVID-19 shot, or any other of the intentions measured.

In Study 2, we investigated whether the results of Study 1 were specific to misinformation or could be generalised to any information about vaccines. Using a design similar as Study 1, the participants were exposed to true headlines supporting or opposing COVID-19 vaccines. The results show that exposure to accurate information, whether pro- or anti-vaccine, did not affect participant intentions. The effect of accurate pro-vaccine information was close to significance, but it *reduced* vaccination intentions, compared to the novel pro-vaccine headlines and the control condition. These surprising results could be because the headlines may have reminded the participants of the growing concerns around vaccine side-effects at the time of the data collection.

Finally, Study 3 explored whether multiple exposures to misinformation would lead to a change in behavioural intentions. Previous research has shown that the strength of intentions to change behaviour following exposure to a fake news headline is significantly correlated with the perceived truthfulness of the headline (Greene & Murphy, 2021). It is therefore possible that participants in Studies 1 and 2 were unconvinced by the headlines and saw no reason to change their behaviour in response. The perceived truthfulness of misinformation can be manipulated experimentally; studies have shown that multiple exposures to a given fake news headline can increase its perceived accuracy (De keersmaecker et al., 2020; Fazio, 2020; Newman et al., 2020; Pennycook et al., 2018). The aim of Study 3 was therefore to increase the perceived accuracy of the headlines via multiple exposures—creating a so-called illusory truth effect (Hasher et al., 1977)—and then to evaluate whether this change would affect behavioural intentions. This experiment therefore compared the effects of single and multiple exposures to novel pro- and anti-vaccine misinformation on vaccination intentions. The analyses show that while multiple exposures did increase the perceived accuracy of the false anti-vaccine headlines, none of the experimental conditions substantially affected vaccination intentions.

Pre-existing opinions regarding vaccination against COVID-19 were controlled for statistically. Additional analyses on the data, looking at the effects of pre-existing opinions on the rates of reported memories for true and false headlines, are reported in a separate paper (Greene et al., 2022). Preregistration included each study plan, hypotheses, sample size, exclusions and analyses. Analyses that were preregistered as exploratory are clearly presented as such. All measures, manipulations and exclusions are reported, and sample sizes were determined via power analysis prior to any data analysis. All materials, data and R scripts are available at https://osf.io/jw23x/.

## Study 1

Study 1 aimed to evaluate the effects of one-shot exposure to misinformation about COVID-19 vaccines on behavioural intentions, including the intention to get vaccinated. We hypothesised (1) that exposure to a fake news story that was *negative* about the COVID-19 vaccine would decrease intentions to be vaccinated, relative to exposure to a positive story or neutral stories; and (2) that exposure to a fake news story that was *positive* about the COVID-19 vaccine would increase intentions to be vaccinated, relative to exposure to a negative story or neutral stories.

### Preregistration

This study was preregistered at https://aspredicted.org/CYW_6RK. Ethical approval was obtained from the University College Dublin Human Research Ethics Committee.

### Participants

Participants were recruited via the platform Prolific and told they were taking part in a study about media exposure and the COVID-19 pandemic. Data were collected between June 8 and June 17, 2021 and included participants from six predominantly English-speaking countries; viz. the UK, Canada, Ireland, USA, Australia, and New Zealand. Prolific allows the screening of participants based on their answers to previous surveys, which was used to select participants by geographical location. This also allowed us to screen out people already vaccinated against COVID-19—although they were asked again in the survey to ensure their status had not changed—and to select participants who had previously provided their opinion on COVID-19 vaccination. In total, 1608 people took the survey, but in line with our preregistration 339 participants were removed: 38 failed an attention check, 302 had received at least one dose of COVID-19 vaccine, and 3 refused a post-debrief consent (with some participants falling in several categories). As preregistered, the final sample included 1269 participants (810 females, 448 males, 11 others; *M* age = 28.54, SD 8.80), which provided 90% power to detect effects of size *f* = 0.1 in a one-way ANOVA.

### Design

This was a between-subjects design, where participants were randomly assigned to one of three exposure conditions: (1) pro-vaccine misinformation, (2) anti-vaccine misinformation, and (3) control (no misinformation). Participants in the pro- and anti-vaccination conditions viewed one fabricated news headline that either supported or rejected COVID-19 vaccination, along with two neutral true stories. Participants in the control condition were exposed to three accurate and neutral headlines.

### Materials

#### Fabricated stories

The fabricated headlines were created for the purpose of this study and were novel to the participants, in order to avoid confounding factors related to previous exposure to the headlines. They aimed to mimic the type of false information that could be found online during that period. Online searches were conducted for each headline, to ensure that they were indeed novel and false. They were piloted between March and June 2021 (*n* = 239), asking participants how plausible they thought each headline was and how likely they thought someone exposed to that headline would be to get vaccinated. The aim was to select the fabricated stories that were most likely to affect behaviour—namely the anti-vaccine stories that scored the lowest for the vaccine intentions and the pro-vaccine stories that scored the highest—and to have balanced plausibility scores between the conditions. Ten headlines were thus selected, five anti-vaccine (e.g. “COVID cover-up: Pfizer ex-employee who was “about to blow the whistle” on the unreported dangerous side effects of the COVID-19 vaccine was involved in a fatal crash. Police are treating the incident as suspicious”) and five pro-vaccine (e.g. “New study finds risk of lung cancer to be significantly reduced after two shots of COVID-19 vaccine”). Each headline was accompanied by stock image of a vaccine vial, presented in “Appendix 1”. All the selected headlines can be found in “Appendix 2”.

#### Neutral true stories

Five neutral headlines about COVID-19 were also created, all describing actual events (e.g. “Tom Hanks was one of the first celebrities to contract COVID-19 back in March of 2020, and now encourages everyone to do their part in preventing the spread of the virus.”) and matched with a relevant photograph (in the case above, a picture of Tom Hanks and his wife). To ensure that the neutral headlines would not have an indirect effect on the behavioural intentions, news related to the state of the pandemic (e.g. as improving or worsening), measures taken (e.g. start of a new lockdown) or the effects of the disease (e.g. long-term effects) were avoided. All the selected headlines can be found in “Appendix 2”.

#### COVID-19 vaccine attitudes

In order to avoid tipping participants off as to the aim of the experiment, we obtained participants’ existing opinions about COVID-19 vaccines from information they had previously provided to Prolific. Attitudes were measured with the question “Please describe your attitudes towards the COVID-19 (Coronavirus) vaccines”, to which they could answer: “For (I feel positively about the vaccines)”, “Against (I feel negatively about the vaccines)”, or “Neutral (I don't have strong opinions either way)”. Because some users had revoked their consent to sharing this information, all the analyses involving pre-existing opinions use a sample of *n* = 1217. In this sample, 573 participants had declared that they supported vaccination, 425 that they were neutral about it, and 219 that they were against it.

### Procedure

**Fig. 1:**
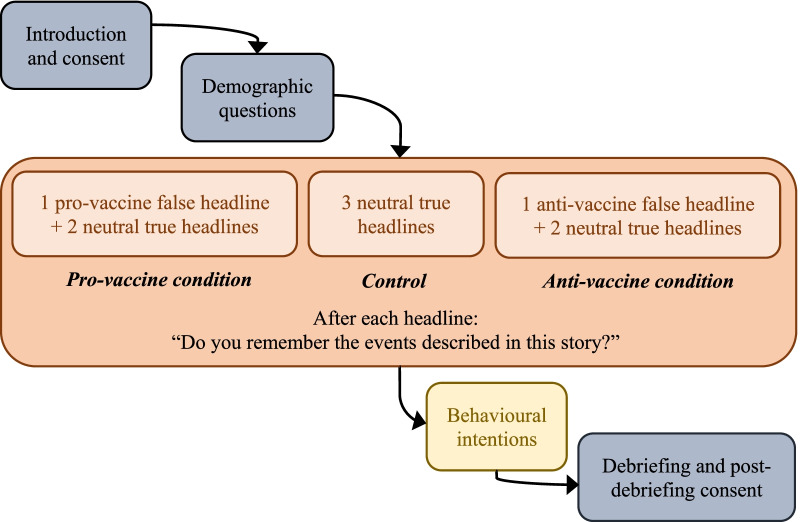
Schematic of the experimental procedure in Study 1

A schematic of the experimental procedure is provided in Fig. 1.

#### Introduction and consent

Participants were informed that the aim of the study was “to investigate reactions to a range of news stories relating to the novel coronavirus outbreak”, with no mention being made of misinformation or fake news. They were then asked whether they consented to take part in the study.

#### Demographic questions

The participants were asked their age, gender, and whether they had received one or more dose of a COVID-19 vaccine.

#### Headlines

They were then shown, depending on the condition and in random order:

- Anti-vaccine condition: one novel anti-vaccine headline and two accurate neutral headlines
- Pro-vaccine condition: one novel pro-vaccine headline and two accurate neutral headlines
- Control condition: three accurate neutral headlines

Each headline was illustrated by an image and presented on a separate page, followed by the question “Do you remember the events described in this story?”. There were four possible answers (I have a clear memory of seeing/hearing about this, I have a vague memory of this happening, I remember this differently, I don't remember this).

#### Behavioural intentions

The participants were asked about their behavioural intentions related to COVID-19 (e.g. “I intend to get a COVID-19 vaccine”), and to a range of unrelated behaviours, specifically exercising more, reducing one’s screen time, getting the COVID-19 vaccine, getting the seasonal flu vaccine, traveling by plane for leisure, giving more money to charity, maintaining social distance, and complying with government mandates. Participants responded to each question on a 7-point Likert scale (1: Strongly disagree; 7: Strongly agree). This series of questions included an attention check (“To show you're not a bot, please select 'strongly disagree' for this question”). All the questions can be found in “Appendix 3”.

#### Debriefing and consent

Finally, participants were taken to a debriefing page, where they were presented again with the headlines and an explanation as to whether they were true or false and why. Following a full debriefing, they were asked if they still consented to participate in the study.

### Results

#### Behavioural intentions

A one-way ANOVA revealed no significant effects of the experimental conditions on vaccination intentions (anti-vaccine: *M* = 4.99, SD = 2.16; control: *M* = 4.96, SD = 2.24; pro-vaccine: *M* = 5.01, SD = 2.18; *F*(2, 1266) = 0.39, *p* = 0.678, *η*_*p*_^2^ = 0.001), and Bayesian analysis indicated strong evidence in favour of the null hypothesis (BF_10_ = 0.03). None of the behavioural intentions, whether related to COVID-19 or not, were significantly affected by the different exposures (see Additional file 1: Table S1 in the supplementary materials for the full results).

**Fig. 2:**
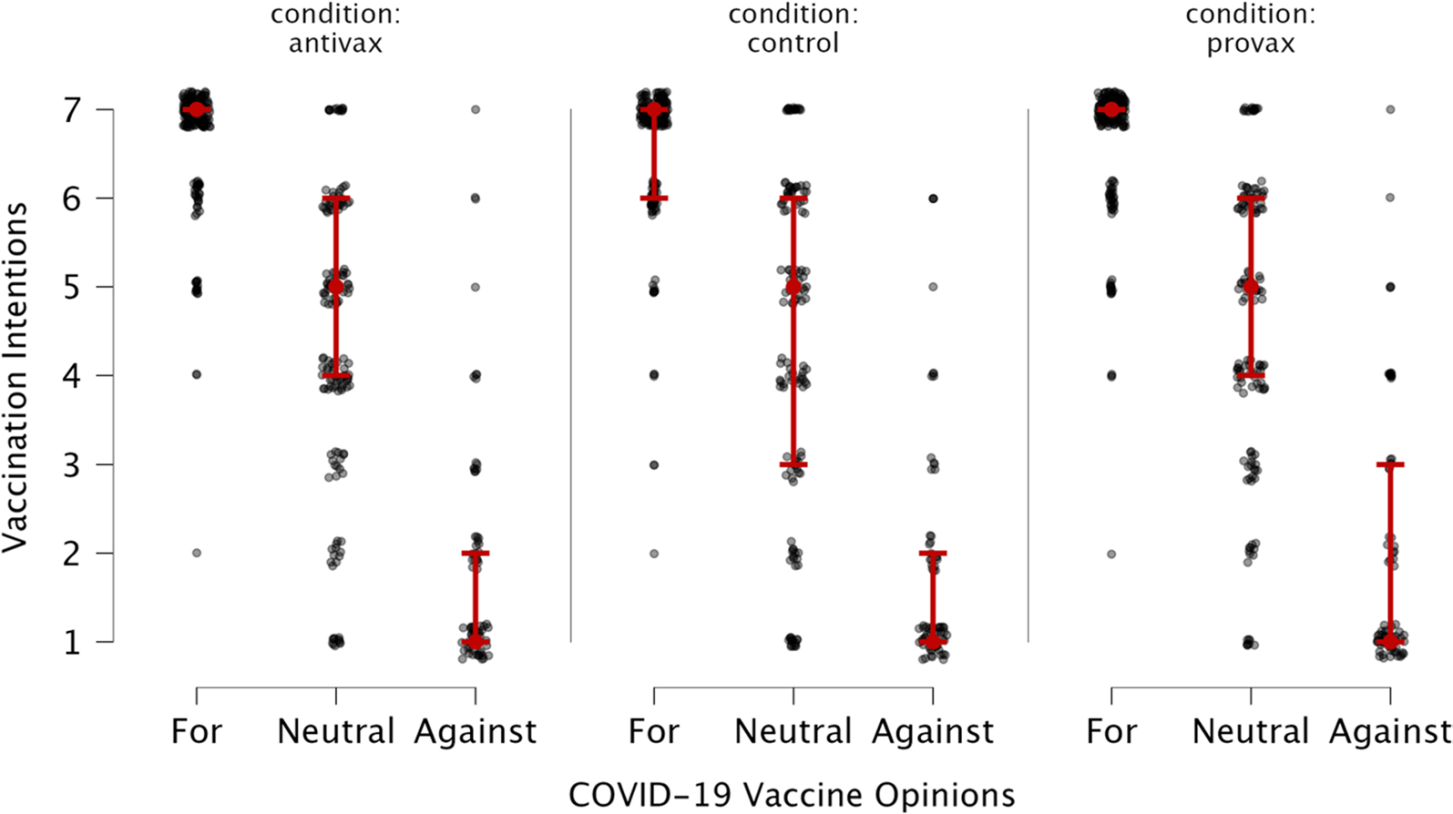
Intentions to get vaccinated in Study 1, by experimental condition and pre-existing vaccine opinions

Overall intentions to get vaccinated against COVID-19 were very high in this sample, perhaps resulting in a ceiling effect. Because of the prevalence of participants who espoused pro-vaccine and neutral attitudes, an ANCOVA was also conducted, controlling for the participants’ pre-existing opinions on COVID-19 vaccines. This analysis was preregistered as exploratory. The results are presented in Table 1 and illustrated in Fig. 2, and are again not significant, although this analysis did increase the effect size.

**Table 1:** Effects of misinformation exposure on vaccination intentions in Study 1, controlling for pre-existing opinions

Pre-existing vaccine opinions	Experimental condition			Group differences
	Anti-vaccine *M* (SD)	Control *M* (SD)	Pro-vaccine *M* (SD)	
Against	1.84 (1.33)	1.78 (1.30)	2.01 (1.47)	*F*(2, 1213) = 1.21, *p* = .300, *η*_*p*_^2^ = 0.002, BF_10_ = 0.03
Neutral	4.40 (1.62)	4.37 (1.87)	4.58 (1.67)	
For	6.64 (0.74)	6.60 (0.81)	6.65 (0.75)	
Adjusted mean	5.00 (2.16)	4.96 (2.23)	4.96 (2.23)	

Vaccination intentions were measured on a scale from 1 to 7, with higher values indicating stronger intentions to get a COVID vaccine

### Study 1 discussion

There are three potential and not mutually exclusive ways of explaining the above results. First, it is possible that the participants had already made up their minds about vaccination based on earlier information, and that attitude change at this stage was difficult. This may be reinforced by the high vaccination intention rate in our sample, creating a ceiling effect for already pro-vaccination participants. Second, our sample size may not have been sufficient to detect a very small effect. If that is the case, it is interesting to note that our results seem to indicate that exposure to both the pro- and anti-vaccine headlines increases vaccination intentions. Reminding people of COVID-19 vaccines may encourage them to get vaccinated. However, our analysis showed an effect size of 0.002, which is negligible in both statistical and practical terms.

Finally, it is possible that the misinformation the participants were exposed to was not sufficient to affect their behavioural intentions. This may be because the information was insufficiently convincing—though it is worth noting that much of the false information circulating on COVID-19 is much more outlandish than the headlines constructed for the study, including, for instance, stories arguing that the vaccines contain 5G microchips that will be used to track or control people. Alternatively, it may be that a single exposure to a piece of information is not enough to affect attitudes, and thus behavioural intentions. To address the possibility that the misinformation we created for this study was simply unconvincing, Study 2 examined the effects of exposure to true news items on vaccination intentions.

## Study 2

Study 2 looked at the effects of exposure to both false and accurate information supporting or rejecting COVID-19 vaccines on vaccination intentions. The design was similar to that in Study 1, but two conditions were added: exposure to accurate pro- or anti-vaccination headlines.^1^ The aim was to investigate whether the lack of significant effect in Study 1 was specific to misinformation, or whether a single exposure to any type of information on COVID-19 would lead to similar results. Data were collected for the two new experimental conditions: exposure to accurate information supporting (true pro-vaccine) or rejecting (true anti-vaccine) COVID-19 vaccination. We hypothesised (1) that exposure to a true or fake *negative* story about the COVID-19 vaccine would decrease intentions to be vaccinated, relative to exposure to neutral stories, and (2) that exposure to a true or fake *positive* story about the COVID-19 vaccine would increase intentions to be vaccinated, relative to exposure to neutral stories.

### Preregistration

This study was preregistered at https://aspredicted.org/Z44_2CG. Ethical approval was obtained from University College Dublin’s Human Research Ethics Committee.

### Participants

Participants were recruited on the platform Prolific and told they were taking part in a study about media exposure and the COVID-19 pandemic. The same selection criteria were applied as in Study 1 (vaccination status and location), and none of the participants had taken part in in Study 1. In addition, the conditions were balanced for pre-existing opinions on COVID-19, with 216 participants for vaccination, 215 neutral, and 215 against. Data were collected between June 18 and June 19, 2021, and 792 people participated. As specified in our preregistration, 146 participants were removed (15 failed the attention check and 134 had received at least one vaccine dose). The final sample included 646 new participants across the two conditions (402 females, 232 males, 12 others; M age = 30.49, SD 10.17), following the sample size per condition in the preregistration.^2^ These participants were compared against the participants from Study 1 for whom pre-existing COVID-19 vaccine opinions were available. The final sample size of *n* = 1863 (n_1_ = 1130 for the analysis of H1 and n_2_ = 1133 for the analysis of H2) provided 80% power to detect effects of size *f* = 0.1 in a one-way ANOVA.

### Design

This was a between-subject design. Newly recruited participants were randomly assigned to one of two exposure conditions: (1) pro-vaccine true information, (2) anti-vaccine true information. These conditions were compared against the misinformation and control conditions from Study 1.

### Materials

#### Accurate headlines

The true headlines were based on accurate information, although the specific phrasing was created for the purpose of this study. They were piloted between March and June 2021, asking participants how plausible they thought each headline was and how likely they thought someone exposed to that headline would be to get vaccinated. The headlines considered the most plausible and which had the most potential to affect behaviour were selected, while ensuring that the scores were balanced across conditions. Ten headlines were chosen, five designed to be anti-vaccine (e.g. “AstraZeneca vaccine advice unlikely to change despite rate of rare clots 'doubling'.”) and five pro-vaccine (e.g. “Pfizer-BioNtech and AstraZeneca jabs effective against 'Indian variant' after two doses”). Each headline was accompanied by a stock image of a vaccine vial. All the selected headlines can be found in “Appendix 4”.

#### Neutral true stories

The study used the same neutral true stories as in Study 1.

#### COVID-19 vaccine attitudes

The participants’ pre-existing opinions on COVID-19 vaccines were collected through the information made available by Prolific, as in Study 1.

### Procedure

The procedure was identical to that employed in Study 1, with the exception of the headlines section. Participants viewed three headlines in random order as follows:

- True anti-vaccine condition: one true anti-vaccine headline and two accurate neutral headlines
- True pro-vaccine condition: one true pro-vaccine headline and two accurate neutral headlines

### Results

All the following analyses were carried out on the pooled data from both Study 1 and 2.

#### Behavioural intentions

The effects of the experimental conditions on vaccination intentions were analysed with two one-way ANCOVAs, controlling for pre-existing COVID-19 vaccine opinions.^3^ Both analyses are presented in Table 2; the first analysis compared the effects of the true and false anti-vaccine headlines against the control group, while the second compared the effects of the true and false pro-vaccine headlines against the control group. The results for the other behavioural intentions are presented in Additional file 1: Table S4 in the supplementary materials.

**Table 2:** Effects of true and false pro- and anti-vaccine information exposure on vaccination intentions in Study 1 and Study 2, controlling for pre-existing vaccine opinions

Pre-existing opinions	News condition				Group differences
	Fake	True	Control	All conditions	
	*M* (SD)	*M* (SD)	*M* (SD)	*M* (SD)	
*Anti-vaccine information*					
Against	1.84 (1.33)	1.82 (1.35)	1.79 (1.30)	1.82 (1.33)	*F*(2, 1126) = 0.21, *p* = 0.813, *η*_*p*_^2^ = 0.003, BF_10_ < .001
Neutral	4.41 (1.62)	4.58 (1.60)	4.37 (1.87)	4.44 (1.71)	
For	6.64 (0.74)	6.57 (0.80)	6.60 (0.81)	6.61 (0.78)	
*Pro-vaccine information*					
Against	2.01 (1.47)	1.71 (1.21)	1.79 (1.30)	1.82 (1.32)	*F*(2, 1129) = 2.87, *p* = .057, *η*_*p*_^2^ = 0.005, BF_10_ = 0.006
Neutral	4.58 (1.67)	4.25 (1.73)	4.37 (1.87)	4.41 (1.76)	
For	6.65 (0.75)	6.56 (1.05)	6.60 (0.81)	6.61 (0.84)	

**Fig. 3:**
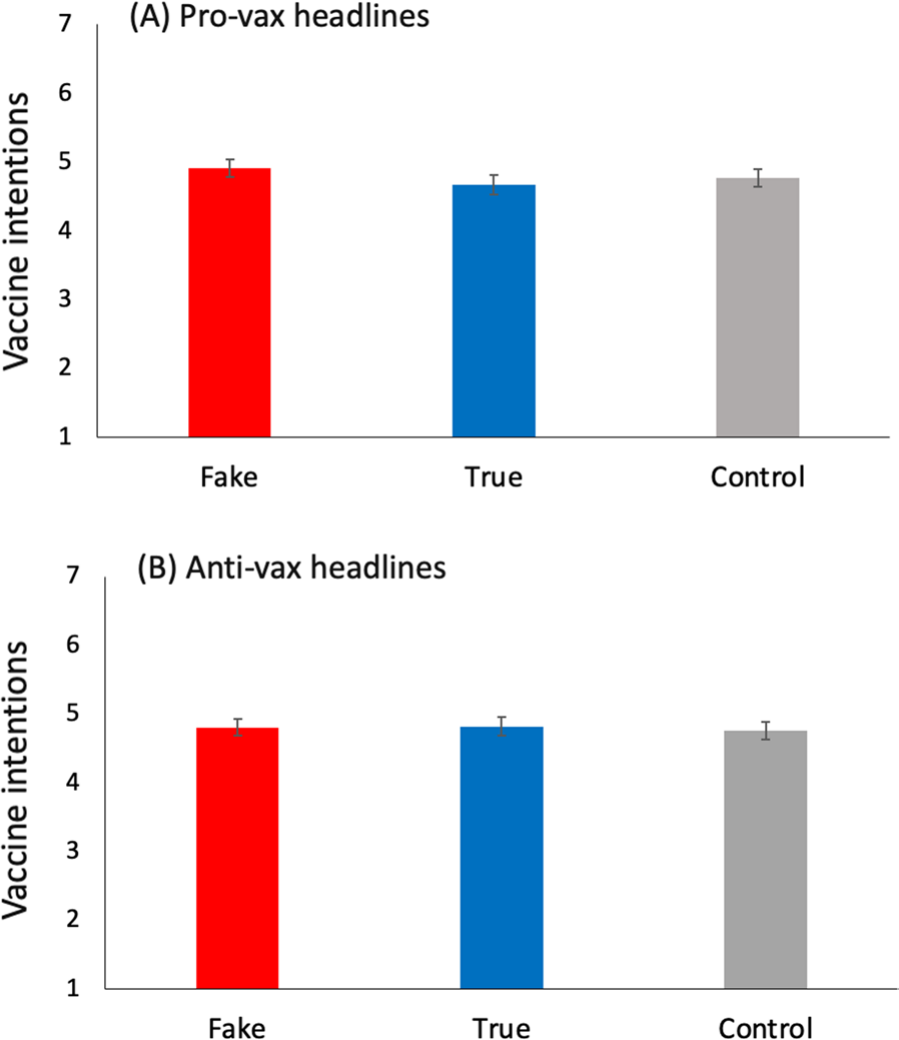
Vaccination intentions in Study 1 and Study 2 following exposure to **A** true and false pro-vaccination headlines and **B** true and false anti-vaccination headlines. Mean values are adjusted for pre-existing vaccine opinions. Error bars represent 95% confidence intervals

Being exposed to anti-vaccine headlines, whether accurate or not, yielded no significant effect on the participants’ vaccination intentions (see Fig. 3B). While the results for the pro-vaccine headlines (Fig. 3A) also failed to reach significance, it was a very narrow miss. For exploratory purposes, post hoc analyses were conducted using Tukey's post hoc tests and revealed that participants in the true pro-vaccine condition were significantly less inclined (adjusted M = 4.67) to receive the vaccine than those in the novel pro-vaccine condition (adjusted M = 4.91, p < 0.001) and control condition (adjusted M = 4.77, p < 0.001). The novel pro-vaccine and control conditions did not significantly differ (p = 0.397).

### Study 2 discussion

Exposure to pro- and anti-vaccine information, whether true or false, did not affect the participants’ intentions to receive a COVID-19 vaccine. This is in line with the results of Study 1 and seems to confirm that the first results obtained are not specific to misinformation. The results for the pro-vaccine conditions, although they fail to reach significance, are a lot more surprising: exposure to accurate information supporting the use of the vaccines seems to have reduced the participants’ intentions to receive a shot. It is possible that although the headlines were designed to support vaccination, which was tested during the piloting phase, they instead reminded the participants of the unknown surrounding side-effects at the time. Indeed, data were collected when the relationship between the Astra-Zeneca vaccine and blood clots was still unclear. The observed effect was small, however, and requires replication.

As with Study 1, it remains possible that the null effects are due to participants only viewing each headline once. Previous research has demonstrated that multiple exposures to a piece of information tend to increase ratings of truthfulness, even for rather implausible items. This repeated exposure lends a sense of familiarity to the items and produces an “illusory truth” effect. In study 3, we investigated whether increasing belief in fake news headlines via multiple exposures would lead to an increased effect on vaccination intentions.

## Study 3

In this study, we compared the effect of a single exposure to novel false information about COVID-19 vaccination with multiple exposures. The design for the participants in the single exposure condition was similar to that in Study 1, with the exception of the question below each headline: instead of asking whether they had seen the headline before, participants were asked to judge how accurate they thought the headline was. The aim of this change was to allow us to measure whether multiple exposures did lead to an increase in perceived accuracy. In the multiple exposure conditions, participants were exposed to the novel headlines twice: first, alongside demographic questions, where they were asked whether they remembered seeing the headlines before, and a second time 3 (± 1) days later, where they were asked to judge their accuracy and to answer the behavioural intentions questions. We addressed our research questions by testing the following formal hypotheses:

- H1: Multiple exposures to a fake news story about the COVID-19 vaccine will increase its perceived accuracy relative to a single exposure.
- H2a: Exposure to a fake news story that is negative about the COVID-19 vaccine will decrease intentions to be vaccinated relative to exposure to neutral stories.
- H2b: Exposure to a fake news story that is positive about the COVID-19 vaccine will increase intentions to be vaccinated relative to exposure to neutral stories.
- H3a: Multiple exposure to a fake news story that is negative about the COVID-19 vaccine with a 3(± 1) days delay will decrease intentions to be vaccinated relative to multiple exposure to neutral stories or to a single exposure to a negative story.
- H3b: Multiple exposure to a fake news story that is positive about the COVID-19 vaccine with a 3(± 1) days delay will increase intentions to be vaccinated relative to multiple exposure to neutral stories or to a single exposure to a negative story.

### Preregistration

This study was preregistered at https://aspredicted.org/B7G_8SP. Ethical approval was obtained from University College Dublin’s Human Research Ethics Committee.

### Participants

Participants were recruited on the platform Prolific and told they were taking part in a study about media exposure and the COVID-19 pandemic. None had participated in Study 1or 2, and all had declared to Prolific that they had not been vaccinated against COVID-19, although they were asked again in the survey. In contrast with the two previous studies, the participants were not screened by geographical location, but the majority came from Europe (66.3%), followed by South Africa (22.2%) and Mexico (7.4%). All the details can be found in Additional file 1: Table S7 in the supplementary materials. Participants were also screened for pre-existing COVID-19 vaccination opinions, to ensure a balance of participants with pro- and anti-vaccine views.

Data were collected between June 24 and June 25, 2021, for the single exposure conditions. For the multiple exposure conditions, data were collected between June 24 and July 2 for the first exposure, and June 27 and July 5 for the second. In total, 2345 people took part in the study: 832 for the single exposure conditions, 1157 for the multiple exposure conditions with 853 returning for the second part (invitations for the second part were closed when the preregistered sample size for phase 2 had been reached), and 178 who exited the survey before being assigned a condition, as they had been vaccinated already. Of the 1685 participants who completed the full survey, and in accordance with our pre-registration, 99 participants were removed for failing the attention check, 17 because they had been vaccinated before the second part of the study, and 22 because they refused the post-debriefing consent. The final sample included 1548 participants (809 men, 719 women and 20 others; M age = 26.46, SD = 7.89). Data on pre-existing opinions on COVID-19 vaccination were available for 1466 participants (589 supporting the vaccines, 531 neutral, and 346 against). This provided 90% power to detect effects of size *f* = 0.1 in a two-way ANOVA.

### Design

This was a between-subject 2 × 3 design, where participants were randomly assigned to one of two exposure conditions: (1) single exposure to the headlines, (2) multiple exposure to the headlines; and to one of three misinformation conditions: (1) pro-vaccine misinformation (2) anti-vaccine misinformation, (3) control (no misinformation).

### Materials

#### Fabricated stories

The fabricated headlines were the same as in Study 1 and can be found in “Appendix 2”.

#### Neutral true stories

Three additional neutral and true stories were added to the five used in Study 1 and Study 2, with a similar procedure. They can be found in “Appendix 5”.

#### COVID-19 vaccine attitudes

The participants’ pre-existing opinions on COVID-19 vaccines were collected through the information made available by Prolific, as in Study 1 and Study 2.

### Procedure

**Fig. 4:**
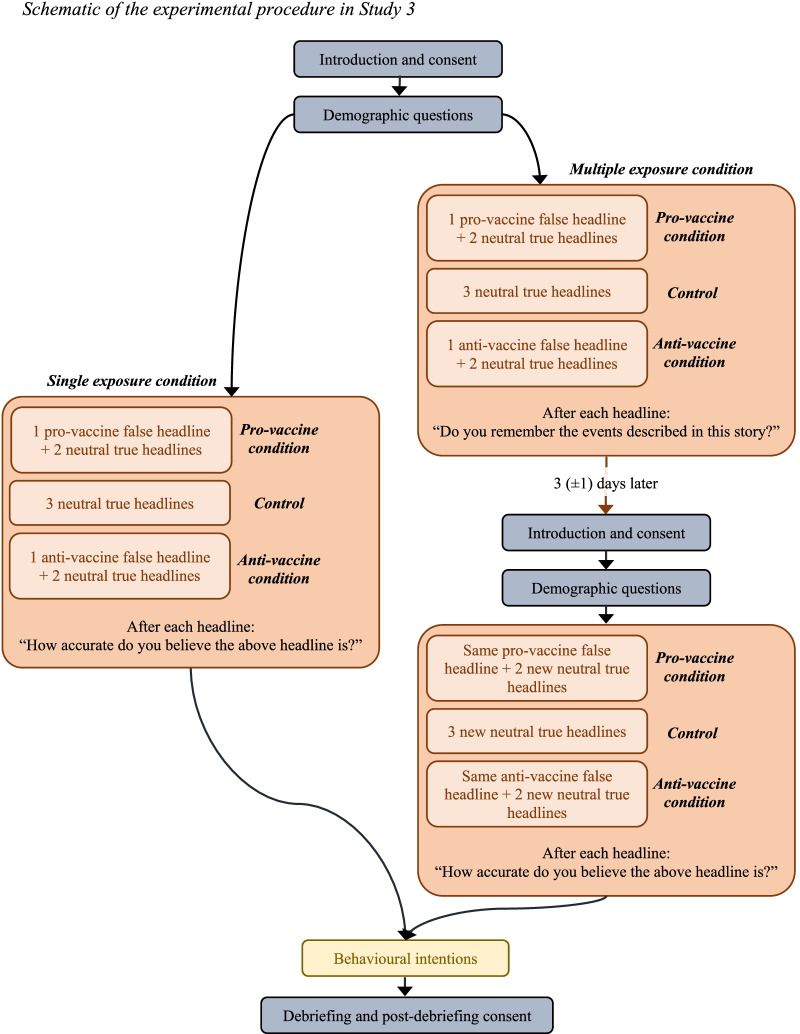
Schematic of the experimental procedure in Study 3

The procedure for Study 3 is outlined in Fig. 4. Participants were randomly assigned to one of two exposure conditions: Single or multiple exposure.

The *single exposure* condition followed the same structure as Study 1: The participants were invited to take part in a survey on COVID-19 and media exposure and asked for consent. They were then asked a few demographic questions and whether they had been vaccinated against COVID-19, before being randomly assigned to one of three misinformation conditions: (1) pro-vaccine, (2) anti-vaccine, and (3) control. They were then shown three headlines in a random order with three between-subjects conditions: (1) one novel pro-vaccine headline and two true neutral headlines, (2) one novel anti-vaccine headline and two true neutral headlines, or (3) three true neutral headlines. However, instead of being asked whether they remembered each headline, they were asked: “How accurate do you believe the above headline is?” alongside a 5-point scale (ranging from “very inaccurate” to “very accurate”, with “I don’t know” as the middle point). Finally, they were taken to the behavioural intentions and debriefing sections.

In the *multiple exposure* condition, the participants took part in two surveys. In the first survey, they were shown the introduction, the consent form, and the demographic questions. They were then assigned to one of three misinformation conditions—pro-vaccine, anti-vaccine, and control—and shown three headlines similar to the single exposure condition. For each headline, they were asked “Do you remember the events described in this story?”, with the same 4-point scale as in the two previous studies. They were then informed that they would be invited to a follow-up study 2 to 4 days later. The second survey followed the same format as the single exposure survey, starting with the introduction, consent, and demographic questions, before taking the participants to the headlines. The anti-vaccine and pro-vaccine headlines shown were the same as in the first survey, but the neutral headlines differed. In other words, participants in the anti-vaccine condition were shown one anti-vaccine headline in the first survey, alongside two true neutral headlines. In the follow-up survey, they were shown the same anti-vaccine headline, and two new true neutral headlines. The same procedure was followed for the pro-vaccine condition. Participants in the control condition were shown three true neutral headlines in the first survey, and three new neutral headlines in the second. After each headline presented in the second survey, they were asked: “How accurate do you believe the above headline is?” alongside a 5-point scale (ranging from “very inaccurate” to “very accurate”, with “I don’t know” as the middle point). All participants were then taken to the behavioural intentions section and the debriefing.

### Results

#### Perceived accuracy

**Fig. 5:**
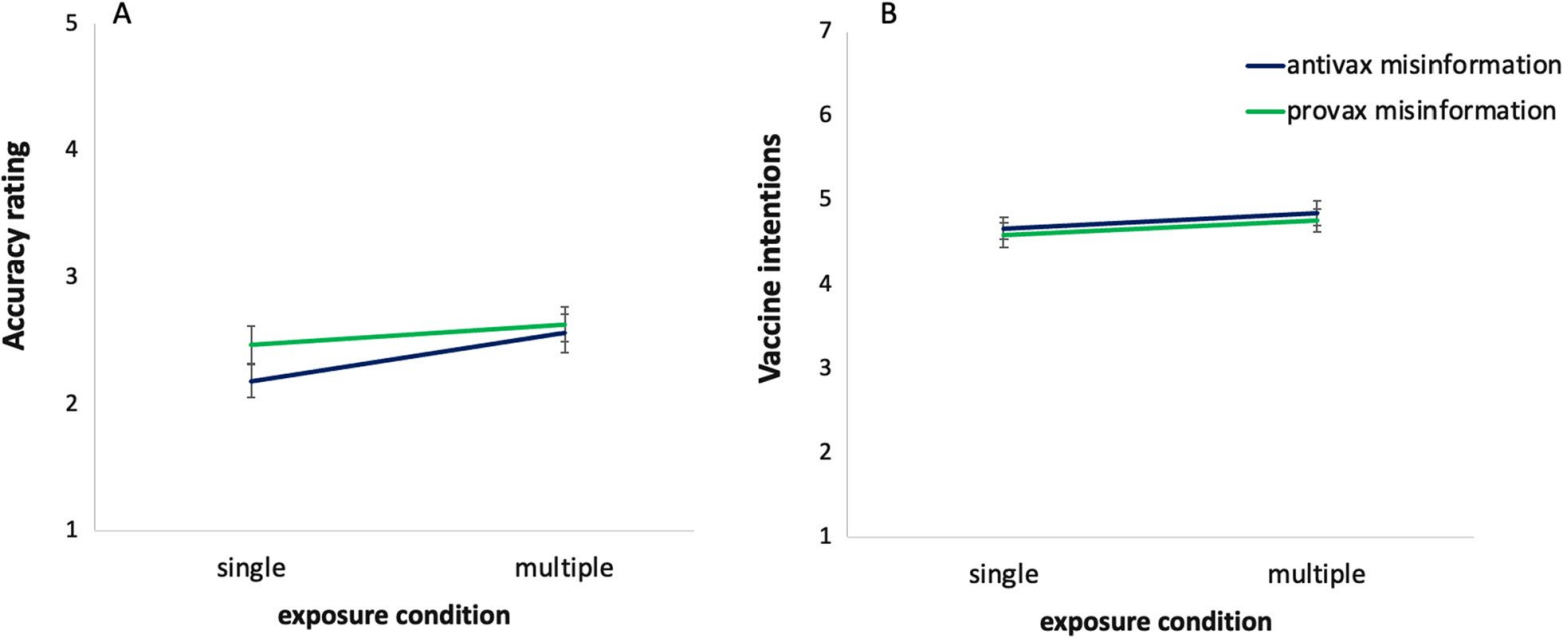
**A** Perceived accuracy and **B** vaccination intentions adjusted for pre-existing vaccine opinions following single or multiple exposure to novel (fake) headlines in Study 3. Error bars represent 95% confidence intervals. Note that while multiple exposures increased accuracy ratings, especially for anti-vaccine misinformation, there was no corresponding effect on vaccine intentions

Two *t* tests were conducted to evaluate the effects of the exposure conditions on the perceived accuracy of the false headlines. The first analysis compared the perceived accuracy of the anti-vaccine headlines and found that the headlines in the multiple exposure condition were rated as significantly more accurate (*M* = 2.56, SD = 1.20) than those in the single exposure condition (*M* = 2.18, SD = 1.07); *t*(509.64) = − 3.80, *p* < 0.001, *d* = 0.33, BF_10_ = 102. The second analysis, comparing the pro-vaccine headlines in the single (*M* = 2.47, SD = 1.21) and multiple exposure conditions (*M* = 2.63, SD = 1.11), failed to reach significance, *t*(504.24) = − 1.59, *p* = 0.11, *d* = 0.14, BF_10_ = 0.36.. Thus, hypothesis 1 was partially supported, and an illusory truth effect was observed following multiple exposures to anti-vaccine misinformation (see Fig. 5A).

#### Behavioural intentions

The effects of the experimental conditions on behavioural intentions were analysed with two-way ANCOVAs, controlling for pre-existing COVID-19 vaccine opinions.^4^ Descriptive statistics are provided in Table 3. As expected, pre-existing vaccine opinions affected vaccine intentions, *F*(1, 1459) = 2023.12, *p* < 0.001, *η*_*p*_^2^ = 0.58. A statistically significant, but very weak effect of misinformation type was observed, *F*(2, 1459) = 3.609, *p* = 0.027, *η*_*p*_^2^ = 0.005. Post hoc tests indicated that while intentions to get vaccinated were slightly higher following exposure to both the pro-vax (*M* = 4.66, SE = 0.065) and anti-vax (*M* = 4.748, SE = 0.064) headlines relative to the control condition (*M* = 4.506, SE = 0.065), the difference was statistically significant only in the pro-vax condition (*t* = 2.648, *p* = 0.022). However, Bayesian analysis indicated moderate evidence for the null hypothesis (BF_10_ = 0.282), suggesting that this effect may be unreliable. There was no effect of exposure condition (single vs. multiple exposures), *F*(1, 1459) = 2.94, *p* = 0.09, *η*_*p*_^2^ = 0.002, BF_10_ = 0.25, and no significant interaction effect, *F*(1, 1459) = 0.38, *p* = 0.68, *η*_*p*_^2^ = 0.001, BF_10_ = 0.002. See Fig. 5B for an illustration. Analysis of other behavioural intentions may be found in Additional file 1: Tables S9 and S10 in the supplementary materials. As in Study 1, none of the analyses reached significance.

**Table 3:** Descriptive statistics of vaccination intentions per exposure (single vs. multiple) and misinformation (novel anti-vaccine vs. control vs. novel pro-vaccine) conditions, adjusted for pre-existing vaccine opinions

Misinformation	Single exposure				Multiple exposures			
	*M*	Adj. *M*	SD	*n*	*M*	Adj. *M*	SD	*n*
Anti-vaccine	4.92	4.66	2.05	244	4.66	4.84	2.23	253
Control	4.52	4.49	2.30	240	4.43	4.52	2.24	248
Pro-vaccine	4.62	4.58	2.25	234	4.70	4.75	2.19	247

### Study 3 discussion

This study partially confirms the presence of an illusory truth effect for COVID-19 misinformation: Anti-vaccine headlines were perceived as more accurate by those who saw them twice than by those who saw them only once. The results were non-significant for the pro-vaccine headlines, but this might the result of a ceiling effect, as (1) the perceived accuracy did change in the expected direction, (2) was close to significance, and (3) these headlines were already evaluated as more truthful than the anti-vaccine ones. It is also worth noting that the perceived accuracy of the false headlines and the participants’ intentions to receive a COVID-19 vaccine were uncorrelated in our dataset (*r*(976) = − 0.02, *p* = 0.610, CI = [− 0.08, 0.05]). More surprisingly, the increase in perceived accuracy did not lead to a clear change in behavioural intentions, nor did the various forms of misinformation about vaccines. If anything, vaccination intentions were higher for those who viewed anti-vaccine headlines when compared to the control group, especially for those exposed to misinformation multiple times. Because this effect was already present in Study 1 and yet failed to reach significance, we conducted an exploratory analysis pooling the data from both studies (*n* = 2683). Using a one-way ANCOVA and controlling for pre-existing opinions of COVID-19 vaccines, we compared the vaccination intentions of the participants in the control group with those exposed to novel misinformation on the vaccines, collapsing across the pro-vaccine and anti-vaccine conditions. Mean vaccination intention (adjusted for pre-existing vaccine opinions) increased slightly, from 4.71 out of 7 (SD = 2.26) in the control condition to 4.86 (SD = 2.17) in the misinformation conditions, *F*(1, 2680) = 7.14, *p* = 0.008, *η*_*p*_^2^ = 0.003, BF_10_ = 0.61.

One possible explanation for this phenomenon is that simply mentioning vaccines primed the participants to think about them, increasing their intentions to get one, although this is inconsistent with the results of Study 2. It is also possible that the anti-vaccine claims backfired: Exposing the participants to antivax rhetoric may have led them to be more critical of those ideas and to adjust their intentions accordingly. In any case, this remains an extremely small effect, supported by Bayes Factors which suggest weak to moderate evidence in favour of the null hypothesis.

The requirement to rate accuracy in Study 3 represents a departure from the method employed in Studies 1 and 2. As noted in the introduction, directing participants’ attention towards accuracy can alter their evaluation and response to information (Pennycook et al., 2020). Nevertheless, this change did not appear to affect participants’ intention to engage in the targeted behaviour, and mean scores on the behavioural intention scale were comparable across all three studies.

## General discussion

It would seem logical to assume that the relationship between misinformation exposure and behavioural intentions is causal and mediated by the perceived accuracy of the false news encountered. In this paper, we have tried to provide empirical evidence for these effects, with no success. In Study 1, we found that exposure to misinformation about COVID-19 vaccines did not affect vaccination intentions as compared to a control group. In Study 2, we found that this effect is not limited to misinformation: a single exposure to accurate information about the vaccines yielded a similar result. In Study 3, we compared the effects of single and multiple exposures, which allowed us to experimentally manipulate the perceived accuracy of the false headlines. Once more, the different conditions did not affect the participants’ intentions to get a COVID-19 vaccine. On the contrary, an analysis of the pooled data from Study 1 and Study 3 showed that being presented with fake news about vaccines—whether pro- or anti-vaccine—positively affected intentions.

There are four potential explanations for the results obtained in these studies. First, as noted in the research literature on attitudes, *changing* and *forming* an attitude are two different phenomena (Crano & Prislin, 2006). It is possible that by the time data were collected, most participants had already formed an opinion on COVID-19 vaccines, making attitude change difficult. This would mean that misinformation may have varying effects depending on when it occurs in the news cycle, with early fake news stories having more impact than those circulating later. If this were to be confirmed, debunking efforts would be best spent by focusing on emerging news rather than on established topics. Relying on algorithms to remove fake news may then problematic, as they often require time to adapt to a new domain (Janicka et al., 2019). By the time they can effectively remove most misinformation on a new topic on social media, most of the damage might already be done.

Indeed, these findings are in line with sequential accounts of persuasion (Bohner et al., 2008), that posit that early persuasion messages affect how later ones are processed. In particular, studies (Bohner et al., 2003; Pechmann, 1992) have shown that negative information presented at a later stage can reinforce positive attitudes—as may have been the case in the anti-vaccine conditions in Study 1 and Study 3—if it is related to earlier positive messages. For instance, mentioning that an ice-cream was high in calories increased the participant’s positive evaluations of the product when it was presented after arguing that it was a particularly tasty treat (Pechmann, 1992), because we tend to associate high calorie content with pleasure. In our experiments, mentions of side effects could have reminded participants of earlier arguments that the risk/benefit balance of COVID-19 vaccines is in favour of vaccination because it protects against a dangerous disease. How the false and anti-vaccine headlines were processed, then, may have been biased by information previously received and may have reinforced positive arguments about vaccination.

Second, it is possible that only particular sources of misinformation or contexts of exposure lead to behavioural changes. For instance, fake news stories shared by friends may be less likely to be believed than those encountered through participation in a scientific study, but may more profoundly affect behaviours. Indeed, the opinions and behaviours of family and friends can affect perceived norms, which can in turn influence planned and actual behaviours (Ajzen, 1991). Alternatively, the participants may have made assumptions about our positions on vaccines and adjusted their responses accordingly. Social desirability has long been shown to be an issue in psychological research (Edwards, 1953), but misinformation studies can also lead to expressive responding (Schaffner & Luks, 2018) and trolling (Lopez & Hillygus, 2018), because of the political implications of the topics investigated. Ecologically valid experiments could help us mitigate these effects and determine in what context misinformation matters more: Publicly shared information on Twitter, for instance, may be more visible and more frequently discussed than fake news circulating relatively unchecked in private groups on WhatsApp, but misinformation shared by friends in this more private setting may have more significant consequences.

Third, the change in perceived accuracy between single and multiple exposures in Study 3 may not have been enough to change the participants’ intentions. A much larger nudge might be necessary to affect behaviours. Likewise, a wider set of beliefs might need to be changed to result in a practical difference: being led to believe that the COVID-19 increases immunity to other diseases—as one of the novel pro-vaccine headlines implied—might not be enough to affect behaviour if one believes it comes at the cost of dangerous side effects. It is therefore possible that isolated exposures have little practical consequence, and that the danger lies in sustained contact with false news. In this regard, Grinberg et al. (2019) showed that during the 2016 presidential election in the USA, 1% of users were exposed to 80% of fake news. Even more strikingly, 0.1% of users were responsible for 80% of the misinformation circulating on the platform. Although misinformation reaches large parts of the population, specific and over-exposed segments might be the ones truly at risk. General debunking campaigns may thus be unnecessary (as well as being ineffective—see Greene & Murphy, 2021)—and may instead need to be targeted at specific groups. The concentration of false news varies widely depending on the social network studied (Cinelli et al., 2020). Efforts might be best spent on the users of a social network like Gab, for instance, than on YouTube, that has eight times less misinformation.

Finally, the false news presented to the participants may be competing with alternative beliefs, limiting their effects even when taken seriously. Participants may be led to believe that some pharmaceutical companies conspired to misinform the public, as one of the headlines implied, but may still give more weight to WHO advice. Therefore, changing someone’s behavioural intentions might require reaching a tipping point, where enough alternative information has been gathered to reverse one’s initial intentions. Nyhan et al. (2020), for instance, found that fact-checking Trump’s claims during his 2016 campaign did improve the factual knowledge of his supporters, showing that the corrections were taken seriously. It nonetheless had no effect on the participants’ opinions of Trump and their intentions to vote for him.

Even in the light of our findings, it seems unlikely that misinformation does not affect behaviour. What we believe our studies point to, however, is that the relationship between exposure to fake news, perceived accuracy, beliefs, and behaviour is not as straightforward as is often assumed. By focusing primarily on perceived accuracy or sharing intentions, research on misinformation may be missing some important aspects of the phenomenon under investigation. More importantly, debunking efforts may be mistargeted. Given how difficult it is to develop and implement interventions that make a lasting impact, it is paramount to make sure that they are aimed at false news that has real consequences, and at those who are the most affected by it.

### Supplementary Information

**Additional file 1:** Supplemental materials.

## Figures and Tables

**Fig. 6:**
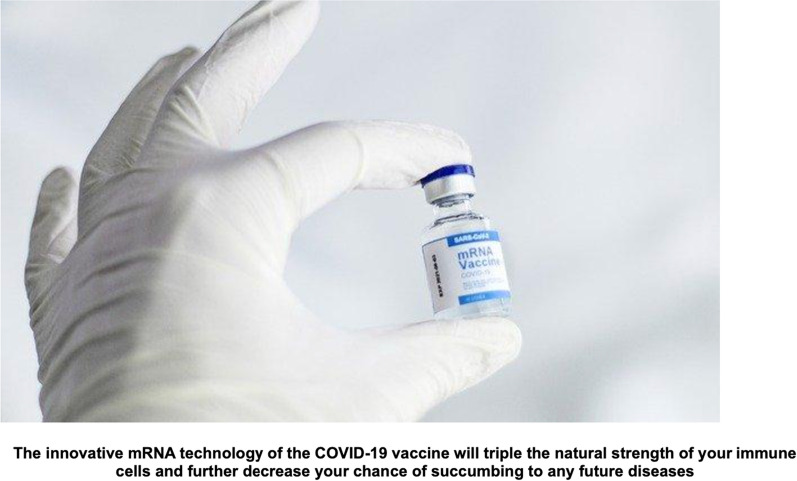
Example of pro-vaccine novel headline

## Data Availability

All data and materials associated with this paper are publicly available at https://osf.io/jw23x/.

## Appendix 1: Examples of vignettes

See Fig. 6.

## Appendix 2: List of headlines in Study 1

### Anti-vaccine novel headlines

1. Episodes of ‘memory loss’ reported after receiving second COVID-19 vaccine dose increased this month.
2. The mRNA technology in the COVID-19 vaccine affects cell mutation and decreases your bone density.
3. The mRNA technology in the COVID-19 vaccine strains your immune cells, making you more susceptible to countless other illnesses.
4. Leaked: In order to maintain the illusion that the pandemic is under control, only 50% of COVID-19 vaccines being administered to the public actually contain the vaccine—the rest are simply placebos
5. COVID cover-up: Pfizer ex-employee who was “about to blow the whistle” on the unreported dangerous side effects of the COVID-19 vaccine was involved in a fatal crash. Police are treating the incident as suspicious.

### Pro-vaccine novel headlines

1. The innovative mRNA technology of the COVID-19 vaccine will triple the natural strength of your immune cells and further decrease your chance of succumbing to any future diseases
2. The mRNA in the COVID-19 vaccine remains in your blood long enough to combat any other flu you may contract in the future
3. Reported ‘side-effects’ associated with the COVID jab are actually caused by vaccine-related anxiety, and not the vaccines themselves—new study finds. The vaccines themselves do not cause any adverse side effects.
4. Regulators were so intent on providing a safe and effective COVID-19 vaccine that the vaccine trials consisted of six phases of testing rather than the usual three.
5. New study finds risk of lung cancer to be significantly reduced after two shots of COVID-19 vaccine

### Neutral true headlines

1. Production for the new Batman movie to be released in 2022 was halted when its star, Robert Pattinson, tested positive for COVID-19
2. The Duke and Duchess of Sussex donated the earnings from the broadcast of their wedding to Feeding Britain U.K. to aid in COVID-19 relief, with a whopping donation of £90,000.
3. Tom Hanks was one of the first celebrities to contract COVID-19 back in March of 2020, and now encourages everyone to do their part in preventing the spread of the virus.
4. In the midst of the pandemic, New Zealand Prime Minister Jacinda Arden’s efforts against COVID-19 were rewarded when she won re-election.
5. After a two-day hospital visit following a positive COVID-19 test, President Donald Trump waved to supporters gathered outside, before heading back to the White House.

## Appendix 3: List of behavioural intentions

1. I intend to get more exercise
2. I intend to reduce my screentime
3. I intend to get a COVID-19 vaccine
4. I intend to get a seasonal flu vaccine
5. I intend to take an airplane journey for leisure purposes, once restrictions are eased
6. I intend to give more money to charity
7. I intend to maintain 'social distance' from others
8. I intend to fully comply with all government mandates regarding COVID-19

## Appendix 4: List of headlines in Study 2

### True anti-vaccine headlines

1. AstraZeneca vaccine advice unlikely to change despite rate of rare clots 'doubling'.
2. Pfizer, Moderna vaccines show limited effectiveness against COVID-19 'Indian variant'.
3. Seychelles, world's most vaccinated nation, faces major COVID spike which suggests limited effectiveness of administered vaccines.
4. Reports of severe, life-threatening allergic reaction (anaphylaxis) occurring after Pfizer COVID-19 vaccine.
5. COVID vaccines associated with false-positive breast cancer result.

### True pro-vaccine headlines

1. COVID-19: First nationwide data from Israel shows 95% protection from infection after two doses of Pfizer jab.
2. Pfizer-BioNtech booster vaccine significantly improves immune responses in patients with cancer.
3. Pfizer-BioNtech and AstraZeneca jabs effective against 'Indian variant' after two doses.
4. Vaccines may provide coronavirus immunity that lasts for years, finds study.
5. Benefits outweigh the risk: Risk of becoming seriously ill from COVID-19 much higher than risk of blood clots from COVID-19 vaccine.

## Appendix 5: List of additional headlines in Study 3

### Additional neutral true headlines

1. Tokyo Olympic organisers approve local spectators amid COVID-19 restrictions but no cheering allowed.
2. Europol: Six arrested in France over suspected COVID benefits scam.
3. COVID-19: Machine that can 'sniff out' coronavirus particles in the air goes on trial in North East of England.

## Footnotes

1 Given the temporal proximity of Study 1 and Study 2 and due to the limited number of suitable unvaccinated participants available on the recruitment platform, additional data were only collected for the new conditions (pro- and anti-vaccine true stories). The analyses presented here were thus conducted on the pooled data from Study 1 and 2. As a result, the hypotheses for the fake news conditions are unchanged from Study 1.

2 Because data for the other conditions were collected in Study 1 and had a larger sample size, the final samples for Study 2 are slightly larger than what was preregistered.

3 Pre-registration planned for two one-way ANOVAs, not controlling for pre-existing opinions. However, the distribution of opinions was unequal between the conditions, affecting the results. The originally planned analyses can be found in the supplementary materials (Additional file 1: Tables S2 and S3), and the analyses presented here were pre-registered as exploratory.

4 Preregistration for Study 3 planned for a two-way ANOVA, with the analysis presented here included as an exploratory analysis. We report here the two-way ANCOVA, controlling for pre-existing opinions, for the sake of consistency with the previous studies. The results of the two-way ANOVA can be found in Additional file 1: Table S8 in the supplementary materials and are similar to the ones presented here.
