# Supplementary material for: Measuring the effects of misinformation exposure and beliefs on behavioural intentions: a COVID-19 vaccination study
Source: Cogn Res Princ Implic. 2022 Oct 1;7:87. doi: 10.1186/s41235-022-00437-y (PMC9526535; doi:10.1186/s41235-022-00437-y)
Supplement: Supplementary file 1 — Additional file 1: Supplemental materials. [file 41235_2022_437_MOESM1_ESM.docx]

**Supplementary materials**

**Study 1**

**Table S.1**

*Effects of misinformation exposure on behavioural intentions, controlling for pre-existing COVID-19 opinions*

|  | Condition | | | | | | | | |  | |  |
| --- | --- | --- | --- | --- | --- | --- | --- | --- | --- | --- | --- | --- |
|  | Fake  anti-vaccine | | | Fake  pro-vaccine | | | Control | | | |  | |
| Dependant variable | M | Adj. M | SD | M | Adj. M | SD | M | Adj. M | SD | | Group differences | |
| Covid Vaccine | 4.96 | 5.00 | 2.16 | 5.10 | 5.09 | 2.14 | 4.98 | 4.96 | 2.23 | | *F*(2, 1213) = 1.21, *p* = .300, η_p_^2^ = 0.002 | |
| Exercise | 5.55 | 5.55 | 1.25 | 5.38 | 5.38 | 1.29 | 5.35 | 5.35 | 1.37 | | *F*(2, 1213) = 2.95, *p* = .053, η_p_^2^ = 0.005 | |
| Screen time | 3.91 | 3.92 | 1.73 | 3.74 | 3.74 | 1.70 | 3.73 | 3.72 | 1.69 | | *F*(2, 1213) = 1.66, *p* = .190, η_p_^2^ = 0.003 | |
| Flu vaccine | 3.36 | 3.38 | 2.02 | 3.33 | 3.32 | 1.94 | 3.30 | 3.29 | 1.93 | | *F*(2, 1213) = 0.30, *p* = .742, η_p_^2^ < 0.001 | |
| Airplane | 4.25 | 4.25 | 2.05 | 4.12 | 4.12 | 2.06 | 4.16 | 4.16 | 2.10 | | *F*(2, 1213) = 0.45, *p* = .636, η_p_^2^ = 0.001 | |
| Charity | 3.65 | 3.65 | 1.48 | 3.49 | 3.34 | 1.55 | 3.52 | 3.38 | 1.50 | | *F*(2, 1213) = 1.48, *p* = .229, η_p_^2^ = 0.002 | |
| Social distance | 4.88 | 4.89 | 1.73 | 5.00 | 5.00 | 1.64 | 5.04 | 5.03 | 1.58 | | *F*(2, 1213) = 0.93, *p* = .393, η_p_^2^ = 0.002 | |
| Government mandates | 5.10 | 5.11 | 1.67 | 5.25 | 5.25 | 1.61 | 5.25 | 5.24 | 1.62 | | *F*(2, 1213) = 1.07, *p* = .344, η_p_^2^ = 0.002 | |

**Study 2**

**Table S.2**

*Effects of anti-vaccine information and misinformation exposure on vaccination intentions in Study 1 and Study 2*

| Fake  anti-vaccine |  | True anti-vaccine |  | Control |  | All conditions |  | Group differences |
| --- | --- | --- | --- | --- | --- | --- | --- | --- |
| M (SD) |  | M (SD) |  | M (SD) |  | M (SD) |  |  |
| 4.96 (2.16) |  | 4.34 (2.34) |  | 4.98 (2.23) |  | 4.79 (2.26) |  | *F*(2, 1127) = 9.15, *p*<.001, η_p_^2^ = 0.02 |

Note: The above table includes the data from both Study 1 and Study 2 to conduct the analyses planned in the pre-registration. However, the sample used in Study 2 was balanced for pre-existing Covid-19 vaccine opinions. The results above are thus affected by these differences. The main manuscript presents the results of a similar analysis – albeit exploratory – controlling for pre-existing opinions.

**Table S.3**

*Effects of pro-vaccine information and misinformation exposure on vaccination intentions in Study 1 and Study 2*

| Fake  pro-vaccine |  | True pro-vaccine |  | Control |  | All conditions |  | Group differences |
| --- | --- | --- | --- | --- | --- | --- | --- | --- |
| M (SD) |  | M (SD) |  | M (SD) |  | M (SD) |  |  |
| 5.10 (2.14) |  | 4.17 (2.40) |  | 4.98 (2.23) |  | 4.79 (2.29) |  | *F*(2, 1130) = 17.99, *p*<.001, η_p_^2^ = 0.03 |

Note: The above table includes the data from both Study 1 and Study 2 to conduct the analyses planned in the pre-registration. However, the sample used in Study 2 was balanced for pre-existing Covid-19 vaccine opinions. The results above are thus affected by these differences. The main manuscript presents the results of a similar analysis – albeit exploratory – controlling for pre-existing opinions.

**Table S.4**

*Effects of misinformation exposure on behavioural intentions, controlling for pre-existing COVID-19 opinions in Study 2*

|  | Condition | | | | | | | | |  | |
| --- | --- | --- | --- | --- | --- | --- | --- | --- | --- | --- | --- |
|  | True  anti-vaccine | | | True  pro-vaccine | | | Control | | | |  |
| Dependant variable | M | Adj. M | SD | M | Adj. M | SD | M | Adj. M | SD | | Group differences |
| Covid Vaccine | 4.34 | 4.60 | 2.34 | 4.17 | 4.45 | 2.40 | 4.98 | 4.54 | 2.23 | | *F*(2, 1042) = 1.04, *p* = .353, η_p_^2^ = 0.07 |
| Exercise | 5.38 | 5.39 | 1.37 | 5.36 | 5.37 | 1.25 | 5.35 | 5.34 | 1.37 | | *F*(2, 1042) = 0.11, *p* = .892, η_p_^2^ < 0.001 |
| Screen time | 3.69 | 3.69 | 1.68 | 3.82 | 3.83 | 1.67 | 3.73 | 3.72 | 1.69 | | *F*(2, 1042) = 0.61, *p* = .541, η_p_^2^ = 0.001 |
| Flu vaccine | 2.96 | 3.14 | 1.88 | 3.01 | 3.09 | 1.93 | 3.30 | 3.09 | 1.93 | | *F*(2, 1042) = 0.11, *p* = .97, η_p_^2^ = 0.008 |
| Airplane | 4.07 | 4.08 | 2.09 | 3.88 | 3.89 | 2.05 | 4.16 | 4.14 | 2.10 | | *F*(2, 1042) = 1.41, *p* = .245, η_p_^2^ = 0.003 |
| Charity | 3.44 | 3.48 | 1.68 | 3.48 | 3.52 | 1.55 | 3.53 | 3.46 | 1.50 | | *F*(2, 1042) = 0.14, *p* = .869, η_p_^2^ = 0.001 |
| Social distance | 4.85 | 4.92 | 1.74 | 4.71 | 4.79 | 1.79 | 5.04 | 4.91 | 1.58 | | *F*(2, 1042) = 0.69, *p* = .500, η_p_^2^ = 0.007 |
| Government mandates | 4.91 | 5.02 | 1.86 | 4.92 | 5.04 | 1.80 | 5.25 | 5.05 | 1.62 | | *F*(2, 1042) = 0.04, *p* = .964, η_p_^2^ = 0.01 |

**Study 3**

**Table S.7**

*Geographical location of the participants in Study 3*

| Country | Number of participants |  | Country | Number of participants |
| --- | --- | --- | --- | --- |
| Australia | 7 |  | Korea | 2 |
| Austria | 7 |  | Latvia | 2 |
| Belgium | 4 |  | Mexico | 115 |
| Canada | 8 |  | Netherlands | 14 |
| Chile | 3 |  | New Zealand | 4 |
| Czech Republic | 17 |  | Norway | 2 |
| Denmark | 9 |  | Poland | 161 |
| Estonia | 17 |  | Portugal | 397 |
| Finland | 4 |  | Slovenia | 15 |
| France | 12 |  | South Africa | 343 |
| Germany | 17 |  | Spain | 83 |
| Greece | 70 |  | Sweden | 9 |
| Ireland | 8 |  | Switzerland | 3 |
| Israel | 1 |  | UK | 93 |
| Italy | 70 |  | USA | 22 |
| Japan | 5 |  | N/a | 11 |

**Table S.8**

*Two-way ANOVA summary for vaccination intentions per exposure and misinformation conditions, for Study 3*

| Source | SS | df | F | *p*-value | η_p_^2^ |
| --- | --- | --- | --- | --- | --- |
| Exposure (E) | 8.3 | 1 | 1.69 | .194 | <0.001 |
| Misinformation (M) | 21.0 | 2 | 2.15 | .117 | 0.003 |
| E x M | 7.2 | 2 | 0.73 | .480 | 0.001 |
| Error | 7138.3 | 1460 |  |  |  |

**Table S.9**

*Effects of misinformation exposure on behavioural intentions in Study 3, controlling for pre-existing COVID-19 opinions*

|  |  | Orientation | | | | | | | | |  |
| --- | --- | --- | --- | --- | --- | --- | --- | --- | --- | --- | --- |
|  |  | Fake  anti-vaccine | | | Fake  pro-vaccine | | | Control | | | |
| Dependant variable | Exposure | M | Adj. M | SD | M | Adj. M | SD | M | Adj. M | SD | |
| Exercise | Single | 5.44 | 5.43 | 1.35 | 5.34 | 5.34 | 1.46 | 5.47 | 5.47 | 1.24 | |
|  | Multiple | 5.31 | 5.31 | 1.36 | 5.43 | 5.43 | 1.42 | 5.42 | 5.42 | 1.30 | |
| Screen time | Single | 3.63 | 3.63 | 1.67 | 3.61 | 3.61 | 1.56 | 3.76 | 3.76 | 1.66 | |
|  | Multiple | 3.92 | 3.92 | 1.77 | 3.74 | 3.74 | 1.61 | 3.97 | 3.97 | 1.71 | |
| Flu vaccine | Single | 3.62 | 3.51 | 1.99 | 3.46 | 3.44 | 1.99 | 3.19 | 3.18 | 1.91 | |
|  | Multiple | 3.22 | 3.29 | 1.90 | 3.23 | 3.25 | 1.98 | 3.37 | 3.41 | 1.89 | |
| Airplane | Single | 4.24 | 4.24 | 1.89 | 3.94 | 3.94 | 2.04 | 4.00 | 4.00 | 1.99 | |
|  | Multiple | 4.12 | 4.12 | 2.10 | 4.11 | 4.11 | 2.12 | 4.00 | 4.00 | 2.03 | |
| Charity | Single | 3.80 | 3.77 | 1.57 | 3.61 | 3.61 | 1.65 | 3.52 | 3.52 | 1.53 | |
|  | Multiple | 3.58 | 3.60 | 1.50 | 3.56 | 3.56 | 1.69 | 3.60 | 3.61 | 1.59 | |
| Social distance | Single | 5.18 | 5.12 | 1.69 | 5.10 | 5.09 | 1.81 | 4.91 | 4.91 | 1.78 | |
|  | Multiple | 5.11 | 5.16 | 1.84 | 5.11 | 5.12 | 1.81 | 5.15 | 5.18 | 1.77 | |
| Government mandates | Single | 5.25 | 5.14 | 1.69 | 5.16 | 5.15 | 1.76 | 5.14 | 5.13 | 1.74 | |
|  | Multiple | 5.05 | 5.12 | 1.81 | 5.23 | 5.25 | 1.84 | 5.17 | 5.21 | 1.77 | |

**Table S.10**

*Effects of misinformation and types of exposure on behavioural intentions in Study 3, controlling for pre-existing COVID-19 opinions*

|  |  | Group differences | | | |
| --- | --- | --- | --- | --- | --- |
| Dependant variable | Independent variable | df | F | *p* | η_p_^2^ |
| Exercise | Exposure | 1, 1459 | 1.03 | .310 | <0.001 |
|  | Orientation | 2, 1459 | 0.58 | .559 | 0.001 |
|  | E x O | 2, 1459 | 0.83 | .438 | 0.001 |
| Screen time | Exposure | 1, 1459 | 3.82 | .051 | 0.004 |
|  | Orientation | 2, 1459 | 0.62 | .540 | 0.002 |
|  | E x O | 2, 1459 | 0.27 | .760 | <0.001 |
| Flu vaccine | Exposure | 1, 1459 | 1.74 | .187 | 0.002 |
|  | Orientation | 2, 1459 | 2.28 | .103 | 0.001 |
|  | E x O | 2, 1459 | 2.35 | .100 | 0.003 |
| Airplane | Exposure | 1, 1459 | 0.41 | .524 | <0.001 |
|  | Orientation | 2, 1459 | 1.46 | .233 | 0.001 |
|  | E x O | 2, 1459 | 0.61 | .544 | 0.001 |
| Charity | Exposure | 1, 1459 | 1.53 | .216 | <0.001 |
|  | Orientation | 2, 1459 | 1.64 | .195 | 0.001 |
|  | E x O | 2, 1459 | 0.88 | .414 | 0.001 |
| Social distance | Exposure | 1, 1459 | 0.07 | .797 | <0.001 |
|  | Orientation | 2, 1459 | 1.03 | .356 | 0.001 |
|  | E x O | 2, 1459 | 0.75 | .472 | 0.001 |
| Government mandates | Exposure | 1, 1459 | 0.01 | .930 | <0.001 |
|  | Orientation | 2, 1459 | 0.01 | .990 | <0.001 |
|  | E x O | 2, 1459 | 0.19 | .830 | <0.001 |
